# Trace element accumulation in bryophytes from Allchar (North Macedonia): contamination or genuine hyperaccumulation?

**DOI:** 10.1007/s00425-026-05092-x

**Published:** 2026-08-05

**Authors:** Ksenija Jakovljević, Marko S. Sabovljević, Katerina Bačeva Andonovska, Lucas Henquet, Antony van der Ent

**Affiliations:** 1 Department of Ecology, Institute for Biological Research Siniša Stanković - National Institute of the Republic of Serbia, University of Belgrade, Belgrade, Serbia; 2 Institute of Botany and Botanical Garden, Faculty of Biology, University of Belgrade, Belgrade, Serbia; 3 Department of Plant Biology, Institute of Biology and Ecology, Faculty of Science, Pavol Jozef Šafárik University in Košice, Košice, Slovakia; 4 Center of Plant Biotechnology and Conservation (CPBC), Belgrade, Serbia; 5 Research Center for Environment and Materials, Macedonian Academy of Sciences and Arts, Skopje, North Macedonia; 6 Laboratory of Genetics, Wageningen University and Research, Wageningen, The Netherlands

**Keywords:** Arsenic, Elemental localisation, Mosses, Thallium

## Abstract

**Main conclusion:**

**Genuine accumulation of metals/metalloids in bryophytes is limited and highly susceptible to surficial contamination with soil particles. Washing with an apolar solvent removes most surficial contamination prior to elemental analysis.**

**Abstract:**

Bryophytes are often the first colonisers of soils toxic from metals/metalloids derived from natural mineralisation or mining wastes. They are ostensibly highly tolerant to the prevailing high concentrations of metals and metalloids in the substrate, but little is known about their ability to (hyper)accumulate these metals/metalloids. Terrestrially growing bryophytes were collected from arsenic-thallium mineralised soils at the Allchar site in North Macedonia. Samples were analysed for elemental concentrations using monochromatic X-ray fluorescence analysis (MXRF) after stringent washing with an apolar solvent and subjected to synchrotron micro-X-ray fluorescence (µXRF) elemental imaging. Scanning electron microscopy (SEM) and energy dispersive X-ray spectroscopy (EDS) were additionally used to assess extraneous contamination and test the efficiency of the washing procedure. The results show that surficial contamination with soil particles is a major challenge for assessing metal and metalloid concentrations in (terrestrial) bryophytes from metalliferous soils. Washing with an apolar solvent (hexane) removes most surficial contamination prior to elemental analysis, indicating some potential for elemental accumulation, as found in *Rhynchostegium megapolitanum* for thallium. Genuine accumulation of arsenic and thallium is relatively low despite the ability to grow on extremely arsenic–thallium enriched soils. Measured elemental concentrations in bryophyte samples are strongly affected by the washing procedure, highlighting the importance of appropiate sample preparation.

**Supplementary Information:**

The online version contains supplementary material available at 10.1007/s00425-026-05092-x.

## Introduction

Spore-forming plants, including ferns (pteridophytes), horsetails (equisetophytes), clubmosses (lycophytes), and mosses (bryophytes) have unique adaptations to colonise edaphically and climatically harsh environments, such as those formed after natural and man-made disasters that create “new” bare conditions. They have, therefore, been termed “Disaster Taxa” (Azevedo-Schmidt et al. 2024). This includes their ability to disperse spores over vast distances, regenerate from dormant spore banks, tolerate temperature, humidity and insolation extremes, withstand adverse soil conditions including low or high pH, salinity and toxic metals, and exhibit exceptional resistance to herbivory (Antreich et al. 2016; Aydoğan et al. 2017; Patiño and Vanderpoorten 2018; Herath and McLetchie 2025). The high tolerance of bryophytes not only enables survival in extreme habitats, but also suggests wider distribution ranges compared to vascular plants (tracheophytes) (Patiño and Vanderpoorten 2018). Moreover, in contrast to tracheophytes, which have a dominant diploid sporophyte, bryophytes are unique among 'Disaster Taxa' in lacking vascularized tissues for mineral nutrient uptake and translocation and possessing a dominant haploid gametophyte (Yadav et al. 2023).

Bryophytes, the second largest group of terrestrial plants, consisting of mosses, liverworts and hornworts, have adapted to various environmental challenges over the course of evolution, enabling them to promote ecosystem succession and recovery, and positive interactions, such as facilitation (Rehm et al. 2019; Qiao et al. 2025). This pioneering role makes them a model system for studying the management of trace elements, especially on mine wastes strongly enriched in metals/metalloids such as copper (Cu), cadmium (Cd), zinc (Zn) and lead (Pb), but also thallium (Tl) and arsenic (As). While the phenomenon of hyperaccumulation in vascular plants is well studied, it is still largely unexplored in bryophytes. Reports of exceptional accumulation of trace elements in bryophytes include, for example: *Scopelophila cataractae* (Mitt.) Broth., a hyperaccumulator of Cu (up to 14,900 µg g⁻^1^), Pb (2390 µg g⁻^1^), and Zn (3690 µg g⁻^1^), including 450 µg g⁻^1^ of As (Suzuki et al. 2016), *Warnstorfia fluitans* (Hedw.) Loeske, an aquatic bryophyte with 4500 µg g⁻^1^ As in its tissues (Sandhi et al. 2018), and *Marchantia polymorpha* L., which accumulates Cd, Cu and Zn under experimental conditions (Ares et al. 2018). Due to the specific morphology of liverworts and predominantly thalloid structure, the ventral part and hyaline parenchyma of *M*. *polymorpha* are in contact with the substrate, causing the basal surface to be highly influenced by the substrate elemental composition (Ares et al. 2018; Degola et al. 2014). In mosses, however, particularly those that are pleurocarpous, prostrate, matted and highly branched, their form makes them predominantly affected by atmospheric deposition, although there is some variation between species (Fabure et al. 2010). Whereas localisation of As in *W. fluitans* remains unknown, in *M. polymorpha* the lower epidermis and hyaline parenchyma are the primary sites for localisation, thus protecting the upper epidermis with function in photosynthesis (Ares et al. 2018). The hyaline parenchyma, a non-photosynthetic tissue with large vacuoles, was also shown to be the main localisation site for Pb in liverwort thallus of *Lunularia cruciata* (L.) Dumort. ex Lindb. (Carginale et al. 2004) and cauloid of moss *Funaria hygrometrica* Hedw. (Basile et al. 2001). With concentrations up to 2000 × higher than in the sporophytes and no Pb detected in the spores and capsules, accumulation in the gametophyte was shown to be the main mechanism of detoxification with sequestration in the vacuoles and protection of reproductive and photosynthetically active tissues (Basile et al. 2001). Elemental immobilisation can further reduce free ionic forms, thereby lowering damage to the cell (Printarakul and Meeinkuirt 2025). Variations in cation exchange capacity (CEC) also play a role in bryophyte tolerance to high concentrations of metals/metalloids, as lower CEC suggests fewer binding sites and reduced elemental transfer to the cell. This leads to decreased accumulation, particularly in the upper plant parts, as shown in Cu-tolerant *Pohlia drummondii* (Müll.Hal.) A.L. Andrews and *Mielichhoferia elongata* (Hook.) Loeske, whereas in non-tolerant *Physcomitrella patens* (Hedw.) Bruch & Schimp., a much more uniform uptake throughout the plant was observed (Antreich et al. 2016).

Bryophytes are an important component of the spontaneous flora in metalliferous habitats, as they can retain considerable concentrations of metals and metalloids (Stanković et al. 2018). Positive correlations between elemental concentrations in bryophytes and environment have been observed in a number of species, confirming their value as biomonitors. However, the mechanisms of elemental uptake in bryophytes still remain largely unknown. For a long time it was considered that, unlike vascular plants, bryophytes lack roots, vascular system and cuticles (Lu et al. 2022). This results in uptake of water with dissolved elements across the entire surface, making them unable to avoid adsorption (Sabovljević et al. 2020). Several pathways for elemental uptake in bryophytes are now recognised, accumulation of tiny soil particles on the surface, ion exchange with carboxyl (-COOH), hydroxyl (− OH) or phosphoryl (− PO_4_^3−^) groups and binding metals on the bryophyte surface, entry of these particles into the cell(s) where they can dissolve, and complexation with chelates being the most common (Stanković et al. 2018). Unlike bryophytes, which lack a developed system for controlled symplastic transport and take up elements across their entire surface moving them passively by diffusion, usually over very short distances, in vascular plants uptake occurs at the root level. Trace elements typically move *via* the apoplast until they reach the endodermis, where they switch to the symplastic pathway. This enables selective and controlled uptake of elements and is crucial for transporting trace elements through the endodermis into the xylem for long-distance movement to the shoots (Marschner 2011). More recent studies, however, have demonstrated the presence of a cuticle, epidermal cells, and a form of vascular system in certain bryophyte species, which is largely species-specific (Varela et al. 2023), providing new insights into elemental uptake in this plant group. The elemental accumulation in bryophytes is partly governed by growth form, as documented for acrocarpous and pleurocarpous bryophytes (Stanković et al. 2021). In contrast to acrocarpous bryophytes, which are mostly tufted, largely attached to substrates, and more influenced by substrate characteristics (Sabovljević et al. 2020), pleurocarpous bryophytes are highly branched, loosely attached to the substrate, and therefore less affected by substrate characteristics, with generally minimal intake of elements (Izquieta-Rojano et al. 2018).

Although some bryophytes are considered hyperaccumulators (Nakajima et al. 2012; Xu et al. 2022), there are still no specific criteria for studying this phenomenon in bryophytes. A major factor affecting the interpretation of elemental accumulation in extra- and intracellular sites of bryophytes is contamination with soil particles, as significant differences in elemental concentrations between unwashed and washed samples have been demonstrated (Fernández et al. 2010). These particles can adhere to the bryophyte surface to varying extents, depending on species-specific characteristics, such as morphology, cation exchange capacity, ion competition, and cell wall composition (including presence of sugars, concentrations of uronic and galacturonic acids, and porosity), which largely influence their binding, and can make removal particularly difficult (Ferreira et al. 2009; Krzesłowska 2011; Petschinger et al. 2021). The number of particles retained on the bryophyte surface may be substantial, although it has been shown that they do not accumulate as many pollutants as expected given their high cation exchange capacity (CEC) (Varela et al. 2023). Another methodological issue in studying elemental accumulation in bryophytes is water availability and the intensity of its flow. Water is the main medium through which elements reach bryophytes; however, high-intensity input, such as heavy rainfall, can cause leaching of elements from bryophytes and obscure their true accumulation potential.

The Allchar site, a thallium–arsenic–antimony (Tl–As–Sb) mineralized outcrop and one of the largest Tl deposits in the world, located on Mt. Kožuf in the Republic of North Macedonia, with up to 18 g kg⁻^1^ of thallium (Tl) and 142 g kg⁻^1^ of arsenic (As) in the soil it is considered one of the most toxic sites globally (Đorđević et al. 2021). The extreme geochemical conditions at Allchar, combined with its position on the southern edge of the last glaciation, have led to intensive adaptation and speciation, resulting in a remarkably rich flora and numerous edaphic tracheophyte endemics (Koljanin et al. 2025), many of which show hyperaccumulation. For example, foliar concentrations of Tl in *Viola allchariensis* Beck and *V*. *arsenica* Beck (Violaceae), two steno-endemic species from Allchar, reached up 23,100 µg g⁻^1^ and 58,900 µg g⁻^1^, respectively (Jakovljević et al. 2023), well above the threshold for hyperaccumulation, set at 100 µg g⁻^1^ (Van der Ent et al. 2013, 2021). Moreover, nearly 80,000 µg Tl g⁻^1^ DW in the leaves of *Silene latifolia* Poir. (Caryophyllaceae) from the same area, represents the highest concentration ever recorded in a living organism (Jakovljević et al. 2025).

In contrast to vascular plants, not much is known about the tolerance of bryophytes and other Disaster Taxa at sites such as Allchar, where they emerge as primary colonisers. This study, therefore, aimed to assess elemental accumulation and distribution in bryophytes growing on one of the most toxic substrates in the world, extending knowledge of their hyperaccumulation and initiating similar studies to develop opportunities for their use in biotechnology and the recovery of contaminated sites. Additionally, the analysis of the effects of the washing procedure on elemental accumulation highlights the importance of standardising the sample preparation process.

## Materials and methods

### Collection of bryophyte (and soil) samples in the field

**Fig. 1:**
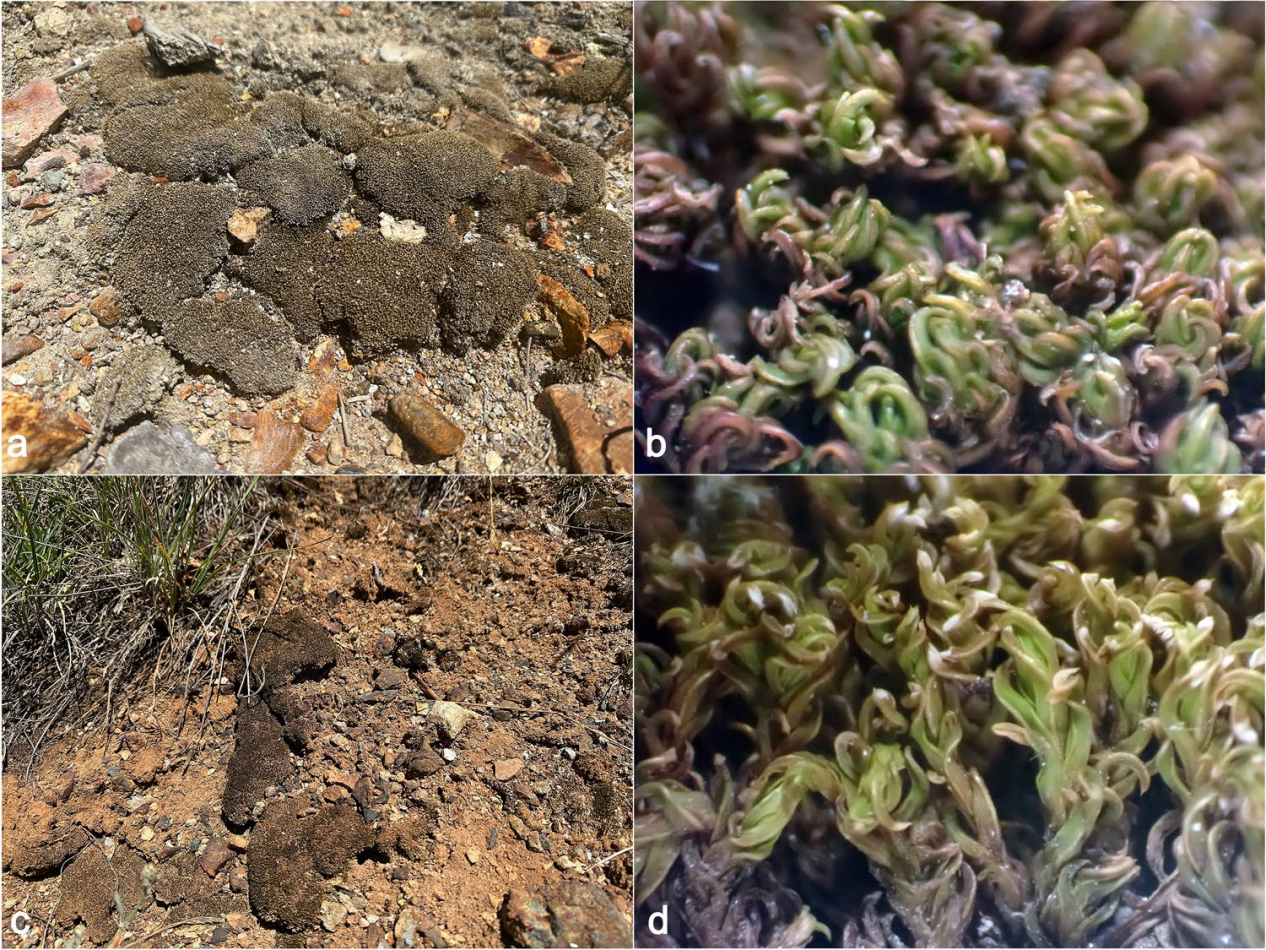
Bryophytes from the Allchar site in North Macedonia. **a**, **b** *Barbula convoluta*. **c**, **d** *Barbula unguiculata*

Bryophytes were collected across the Allchar site, with a sample surface of approximately 10 cm^2^, to ensure a sufficient quantity for analysis without jeopardizing the local population. The samples were collected on the most mineralised soils in the three sublocalities (Crven Dol, Central Deposit, Southern Deposit), differing in their local elemental composition (Jakovljević et al. 2025). The sampling focussed on terrestrially growing bryophyte species, as these are in direct contact with the toxic soil (Fig. 1, Table S1). In total, 40 samples were collected representing 11 taxa. Representative soil samples were also collected for chemical analysis. The bryophyte vouchers are deposited in the BEOU-BRYO collection (Table S1; Index Herbariorum 2026).

### Elemental analysis of bryophytes and soil material samples

The soil samples were collected from the bryosphere, dried and sieved using a 2-mm stainless mesh screen. For MXRF analysis, 0.2 g of each sieved soil subsample was placed in custom MXRF sample holders and covered with 6.0 µm thickness polypropylene thin film (Chemplex Industries Inc.). The soil samples were analysed using a Z-spec JP500 instrument (Z-Spec Inc.) in ‘Soil’ mode (Zhang et al. 2026). Two different certified standards were used (NIST SRM 2702, marine sediments and NIST SRM 2709a San Joaquin soil for soils) for the quality control of the soil material analysis.

The bryophyte material was kept in paper bags during transport to the laboratory and air-dried in the shade at room temperature for 4 days until a constant weight was achieved. Prior to analysis, the plant material was brushed to remove excess soil and split evenly into two samples: one remained unwashed and the other was subjected to a stringent cleaning procedure to remove any surficial dust contamination using hexane, following methods described in the literature for plant material (Reeves and Kruckeberg 2018; Paul et al. 2019). Briefly, dried bryophyte material samples (~ 1 g) were immersed in 20 mL of anhydrous hexane (HPLC-grade, ≥ 95%, Sigma-Aldrich) in 50 mL polypropylene tubes, which were then placed in an ultrasonic bath for 60 s. The samples were carefully removed from the tubes, the hexane was allowed to evaporate from the material, and the thus cleaned samples were dried in a ventilated oven at 60 °C for 48 h. Both washed and unwashed samples were subsequently ground to a fine powder (< 200 µm) in an impact mill (IKA Tube Mill 100 Control) and 0.5 g subsamples were placed into custom XRF sample holders and covered with 6.0 µm thickness polypropylene thin film (Chemplex Industries Inc.) for the MXRF analysis. The powdered plant material was analysed using a Z-Spec JP500 instrument (Z-Spec Inc.) in ‘Plant’ mode (Zhang et al. 2026). This instrument uses monochromatic X-ray fluorescence excitation at 17.48 keV to analyse elements from Z = 13 (Al) to *Z* = 40 (Zr) on the K-lines and up to *Z* = 92 (U) on the L-lines with optimum sensitivity for elements Cu–Se and Hg-Tl-Pb and limits of detection (LODs) ranging from 0.009 to 0.025 µg g⁻^1^. Two certified standards were included for the plant material analysis (NIST SRM 1570a Trace elements in spinach leaves, and NIST SRM 1573a in tomato leaves). The recovery for each of the elements tested was between the 95% and 105%.

### Synchrotron µXRF elemental imaging

The X-ray fluorescence elemental imaging was carried out at PETRA III (Deutsches Elektronen-Synchrotron, DESY, Hamburg, Germany), a 6-GeV synchrotron radiation source, specifically at the hard X-ray microprobe experiment on the undulator beamline P06 (Boesenberg et al. 2016). The P06 beamline is equipped with a cryogenically cooled Si (111) double-crystal monochromator. An ion chamber upstream of the sample is used to monitor the incoming flux, while a 500-μm-thick Si PIPS diode with a 19-mm-diameter active area [PD300-500CB, Mirion Technologies (Canberra) GmbH, Rüsselsheim, Germany] located downstream of the sample records the transmitted X-ray intensity to extract absorption data. A pair of Kirkpatrick–Baez mirrors was used to focus the 16 keV X-ray beam down to about 460 nm × 400 nm (horizontal × vertical) resulting in a flux of about 7.6 × 10^9^ photons s^−1^ on the sample. The XRF signal was detected using a 16-element Ascanio silicon drift detector (Politecnico di Milano) placed in backscatter geometry (180°). All XRF detectors were used in combination with Xspress 3 pulse processors (Quantum Detectors, Didcot, UK). Field collected specimens of selected bryophytes (*Barbula convoluta*, *B*. *unguiculata*, *Hypnum cupressiforme* var. *lacunosum*, *Ptychostomum capillare,* and *Rhynchostegium megapolitanum*; Table S1) were brought in an air-dried state to DESY and revived with demineralised water for 24 h. Individual leaves (phylloids) were extracted and mounted on Kapton sticky tape with silicon adhesive (3 M™ Polyimide Film Tape 5413) stretched over a 3D-printed ABS plastic frame. They were covered with Ultralene® Window Film, 6.0 µm thick (Cole-Parmer SamplePrep 3525; Cole-Parmer, Metuchen, NJ, USA). To further assess the effect of soil particle contamination, *B*. *convoluta* and *P*. *capillare* were analysed in two samples each.

### Scanning electron microscopy (SEM) with energy-dispersive X-ray spectroscopy (EDS)

The analyses were carried out on samples of *B*. *convoluta*. The bryophyte specimens (prepared as described in *Elemental analysis of bryophytes and soil material samples*) were mounted onto aluminium SEM stubs using carbon stickers and sputter coated with 12 nm tungsten (SCD 500, Leica) to ensure good conductivity. The samples were imaged using a field emission SEM (Magellan 400, Thermo Fisher Scientific/FEI) equipped with an EDS detector (Xmax 50, Oxford Instruments). An overview scan of the sample was performed using the SE-detector, with a 13 pA beam current, an acceleration voltage of 2 kV and magnifications of 100–500 × . The EDS mapping used a beam current of 80 pA and an acceleration voltage of 15 kV. The data were visualised with Aztec software (Oxford Instruments).

### Data analyses

Data acquisition was performed using a custom workflow (Garrevoet 2025) and the XRF data were processed with non-linear least squares fitting as implemented in PyMCA (Solé et al. 2007). Energy calibration was achieved by measuring various thin film deposited foils with XRF emission lines that were well-spaced over the energy range (Micromatter Technologies Inc., Surrey, BC, Canada). The matrix used for modelling was hydrated plant material with the empirical formula C_7.3_O_33_H_59_N_0.7_S_0.8_, a density of 0.8 g cm⁻^3^ and a thickness of 300 µm. This resulted in 32-bit.tiff files with pixel values corresponding to the area density of each element in µg cm⁻^2^ converted to µg g⁻^1^ values by: measured area density (μg cm⁻^2^)/(thickness of the layer (cm) × reference volume density (g cm⁻^3^)). The figures were prepared in ImageJ (Schneider et al. 2012) by changing the lookup table (LUT) to ‘Fire’ and adjusting the pixel intensity range with the levels slider so that the background is scaled to black and the highest concentration values are scaled to the upper end of the LUT (nearly white). Concentration scale bars were added using the ‘calibration’ tool, along with length scales.

Elemental concentrations in the samples are presented as the mean, standard deviation, and range. To assess differences in elemental concentrations among species and washing procedures, a two-way ANOVA (statistical significance at *p* < 0.05) was performed, with post hoc comparisons using the Tukey HSD test. The analyses were carried out in RStudio software v. 4.5.2 (R Core Team 2026). The relationship between elemental concentrations in plants before and after washing was analysed using the Spearman correlation coefficient, performed in PAST 4.03 (Hammer et al. 2001).

## Results

### Elemental concentrations in bryophytes and associated soil

Elemental concentrations in the surface soil varied greatly among samples, with generally anomalous concentrations of As and Tl, reaching up to 62,800 µg g⁻^1^ and 3810 µg g⁻^1^, respectively. Considerable differences were also found between sites, with the highest concentrations in samples from the Southern and Central Deposit (Table 1). Elemental concentrations in bryophytes were somewhat lower, being the highest at 25,000 µg g⁻^1^ and 1890 µg g⁻^1^ for As and Tl, respectively. Arsenic concentrations exceeded the threshold for hyperaccumulation in 34 samples, whereas only 13 samples contained Tl at concentrations > 100 µg g⁻^1^, indicating potential hyperaccumulation. Potential simultaneous hyperaccumulation of As and Tl was found in *Ptychostomum capillare* and *Rhynchostegium megapolitanum*, with the latter predominantly hyperaccumulating Tl (up to 1890 µg g⁻^1^). Arsenic concentrations above 1000 µg g⁻^1^ were found in seven taxa, with the highest recorded in a *Barbula convoluta* sample, which was statistically different from other species analysed both before and after washing with hexane (Tables 2 and S2). For Tl, no significant differences were found between species or washing procedures applied.

**Table 1:** Concentrations of arsenic and thallium given as means and standard deviations (in µg g⁻^1^) in soil samples (*n*, number of samples) associated with the collected bryophytes from the Allchar site

Taxon	Locality	*n*	As	Tl
*Barbula convoluta*	Central Deposit	2	58,200 ± 212	3810 ± 1500
	Southern Deposit	1	62,800	2390
*Barbula unguiculata*	Central Deposit	1	12,800	784
*Hypnum cupressiforme*	Crven Dol	1	7350	365
	Central Deposit	1	861	67.8
	Southern Deposit	1	8810	557
*Hypnum cupressiforme* var*. lacunosum*	Crven Dol	1	4930	482
*Ptychostomum capillare*	Southern Deposit	1	55,100	1010
*Rhynchostegium megapolitanum*	Crven Dol	2	11,700 ± 1620	767 ± 89.1
	Central Deposit	4	24,700 ± 16,600	2560 ± 1790
	Southern Deposit	1	10,200	983

**Table 2:** The mean ± standard deviation and range of concentrations of arsenic and thallium (in µg g⁻^1^) in bryophyte taxa (*n*, number of samples) from the Allchar area under different treatments

Taxon	Treatment	*n*	As		Tl	
			Mean ± SD	Range	Mean ± SD	Range
*Barbula convoluta*	Unwashed	5	12,700 ± 11,100^b^	61.4–23,600	6.86 ± 12.4^a^	0–28.5
	Washed	5	13,400 ± 10,400^c^	76.3–25,000	1.15 ± 2.56^a^	0–5.73
*Barbula unguiculata*	Unwashed	2	3540 ± 1840^ab^	220–4840	31.9 ± 45.2^a^	0–63.9
	Washed	2	13,600 ± 6510^bc^	9010–18,200	0^a^	0
*Hypnum cupressiforme*	Unwashed	17	1030 ± 2360^a^	3.84–8060	15.9 ± 24.0^a^	0–83.7
	Washed	16	962 ± 3310^a^	3.49–13,400		0–138
*Ptychostomum capillare*	Unwashed	4	3800 ± 1400^ab^	2410–570	226 ± 452^a^	0–903
	Washed	8	1820 ± 1310^ab^	0–3570	105 ± 269^a^	0–769
*Rhynchostegium megapolitanum*	Unwashed	9	1850 ± 3370^a^	44.4–10,600	399 ± 672^a^	0–1890
	Washed	11	790 ± 1280^ab^	16.3–4370	145 ± 201^a^	0–491

Different letters indicate significant differences according to Tukey’s test (*p* < 0.05) for inter-species comparisons within the same treatment. This is standard for indicating the results of statistical tests

The concentrations of Ti and Cr, indicators of surficial contamination, corresponded to the enrichment of As, one of the dominant elements in soil (ρ_As-Ti_ = 0.72 and ρ_As-Cr_ = 0.55). For Tl, the opposite was observed, with negative correlations with As, Ti and Cr in both unwashed and washed samples (Fig. S1). The efficiency of stringent washing with hexane varied between samples, resulting in reduced elemental concentrations in most of the taxa analysed. The efficiency was particularly high in samples representing *Hypnum cupressiforme* (> 80% of As removed at > 90% of Ti and Cr removed, and up to 80% of Tl removed)*, R. megapolitanum* (up to 60% of As and 100% of Tl removed) and *P. capillare* (> 50% of As removed, with limited uptake of Tl) (Table 3)*.* In samples representing *Barbula* spp. removal of As, Ti and Cr was low, with some showing higher elemental concentrations after washing (Table S3).

**Table 3:** Washing efficiency (percentage of concentration removed) for arsenic and thallium in bryophyte samples (*n,* number of samples) from Allchar, presented as mean ± standard deviation, and minimum and maximum values

Taxon	As			Tl			*n*
	mean ± SD	min	max	mean ± SD	min	max	
*Barbula convoluta*	− 50.7 ± 102	− 232	9.74	20.3 ± 44.6	1.39	100	5
*Barbula unguiculata*	− 289 ± 18.4	− 302	− 276	50 ± 70.7	–	100	2
*Dicranella howei*	37.7	–	–	0	–	–	1
*Homalothecium lutescens*	− 97	–	–	100	–	–	1
*Hypnum cupressiforme*	− 11.6 ± 95.6	-292	81.4	2.84 ± 37.9	− 71.7	45.8	13
*Hypnum cupressiforme* var. *lacunosum*	− 503*	–	–	100*	–		1
*Ptychostomum capillare*	42.9 ± 25.7	− 2.85	76.3	2.97 ± 6.65	–	57.4	5
*Rhynchostegium megapolitanum*	36.0 ± 21.8	− 24.5	58.9	2.53 ± 69.5	− 133	100	8
*Syntrichia ruraliformis*	5.39*	–	–	− 25.0*	–	–	1
*Syntrichia ruralis*	− 17*	–	–	− 55.2*	–	–	1
*Syntrichia subpapilosissima*	25.7*	–	–	23.6*	–	–	1

*The reported value is based on a single sample

### Distribution of selected elements in bryophytes

**Fig. 2:**
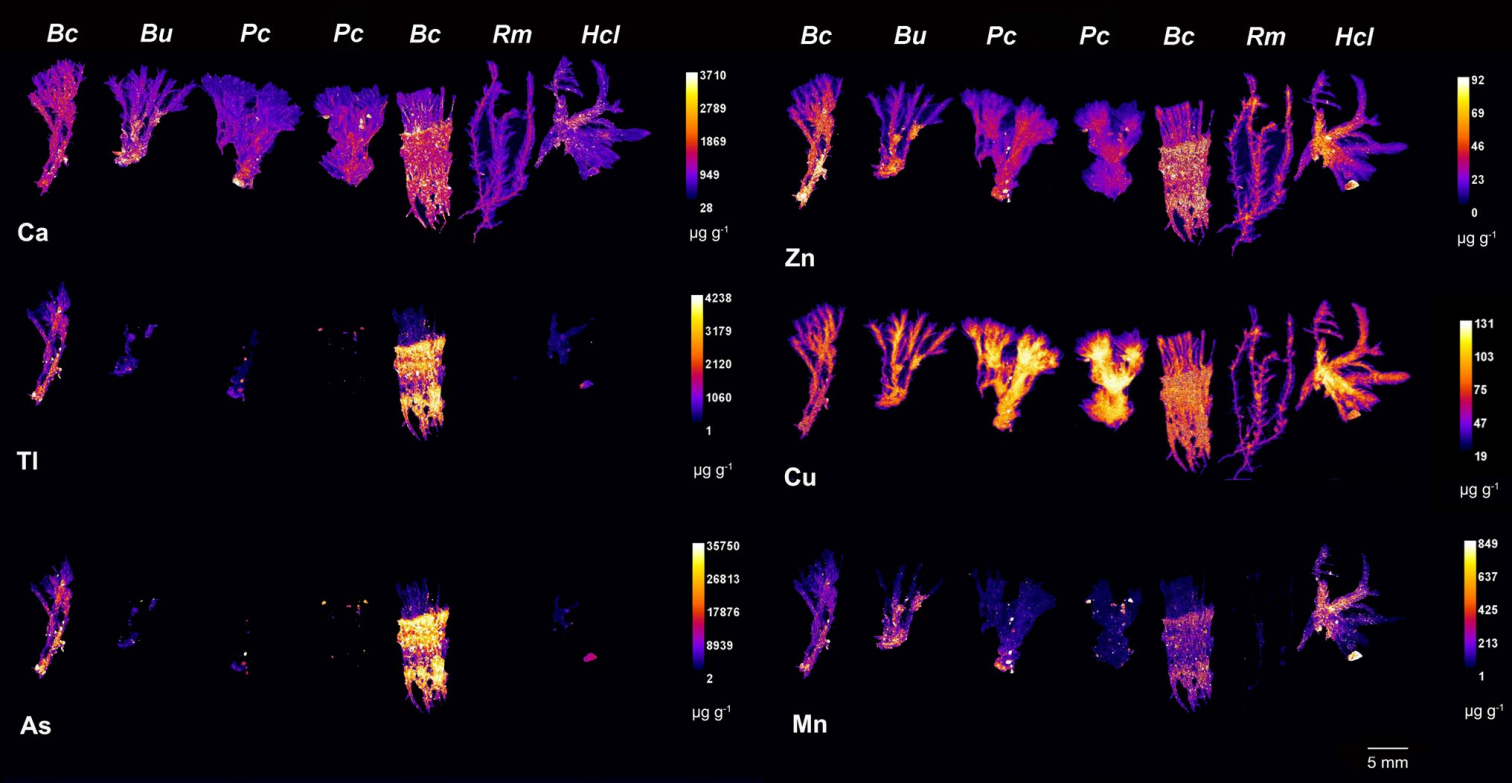
Synchrotron µXRF elemental maps showing the distribution of Ca, Tl, As, Zn, Cu, and Mn in selected bryophyte species (from the left to the right): *Barbula convoluta* (*Bc*), *B*. *unguiculata* (*Bu*), *Ptychostomum capillare* (*Pc*), *P*. *capillare* (*Pc*), *B*. *convoluta* (*Bc*), *Rhynchostegium megapolitanum *(*Rm*), *Hypnum cupressiforme* var. *lacunosum* (*Hcl*)

**Fig. 3:**
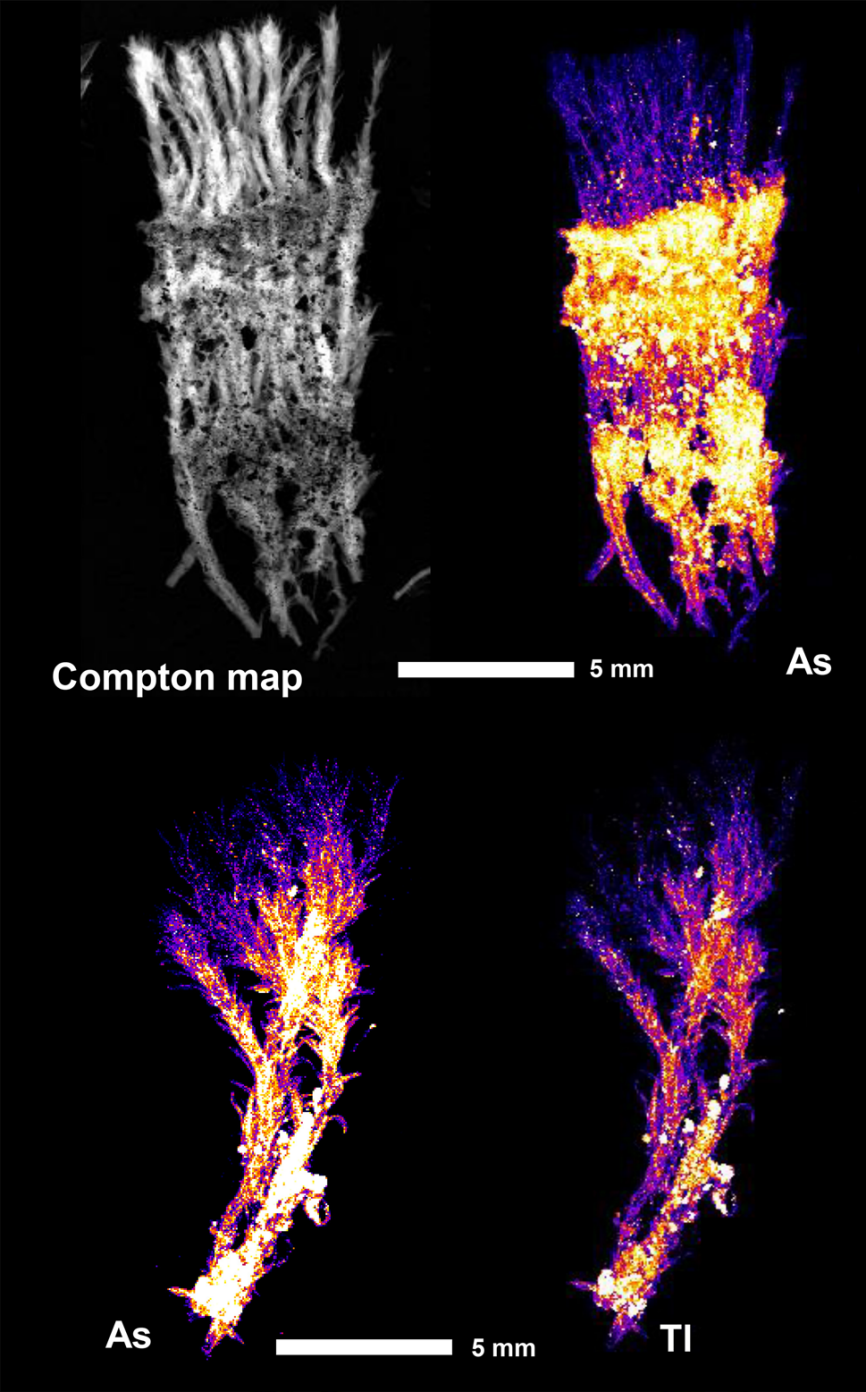
Close-up detail of synchrotron µXRF elemental maps shown in Fig. 2, illustrating the distribution of arsenic and thallium in *Barbula convoluta*

The spatial elemental distribution in selected bryophyte species showed predominant enrichment in the lower parts of both acrocarpous and pleurocarpous species, largely reflecting surficial contamination with soil particles and longer exposure to contaminants (Fig. 2). The concentrations were particularly high for As and Tl, especially in *Barbula convoluta*, being markedly higher compared to the other species analysed. Co-localisation was found for particles rich in As and Tl, with higher intensity for As (in some areas so high that it disrupts reliable measurements) (Fig. 3). Similarly, enrichment was observed for Cu in *P. capillare*, whereas the distribution of Ca, Zn, and Mn was more uniform among specimens.

**Fig. 4:**
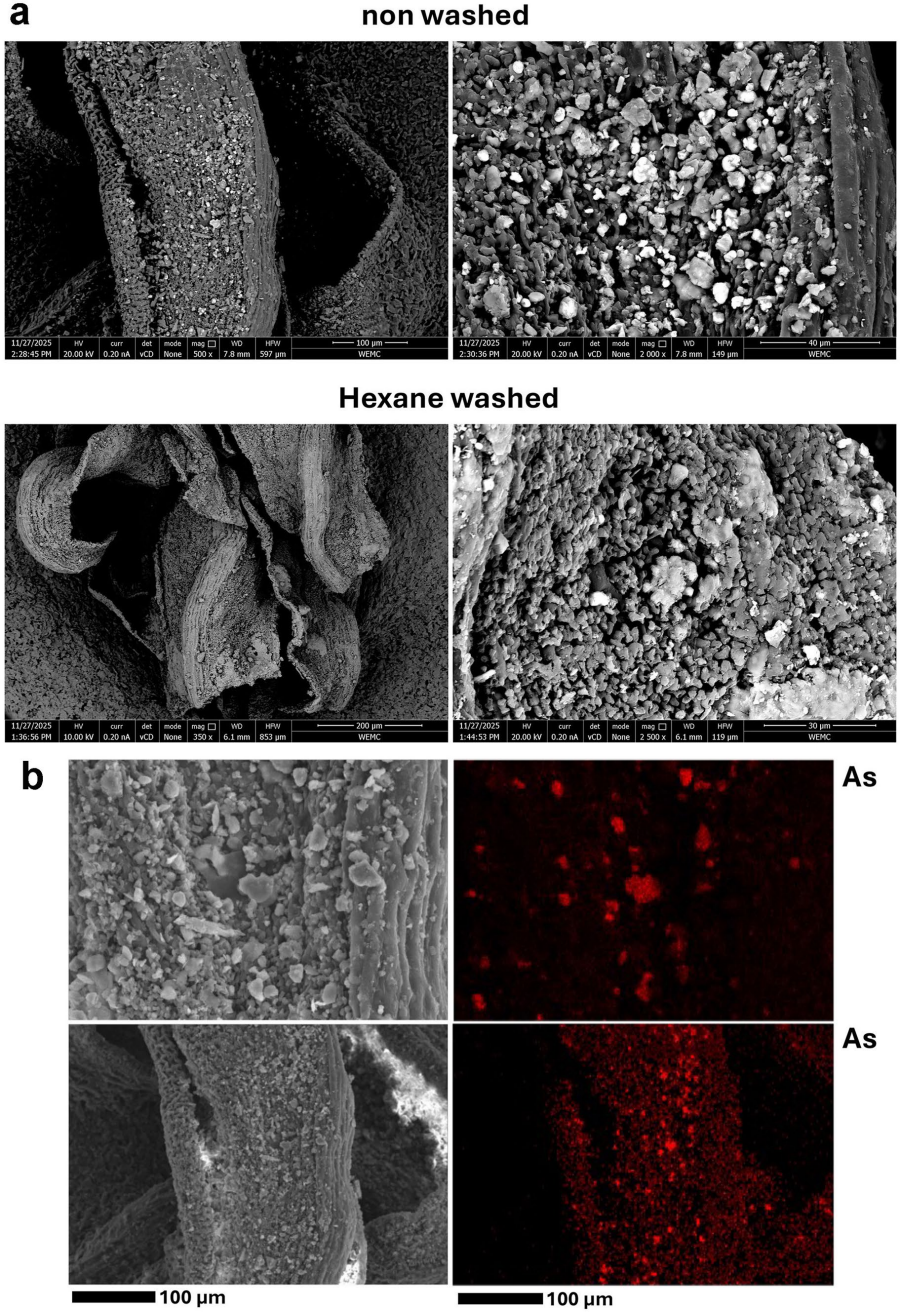
**a** Scanning electron microscopy with EDS showing the distribution of non-washed and hexane-washed samples of *Barbula convoluta.* **b** Distribution of arsenic particles on the *B. convoluta* surface

The SEM–EDS analysis of *B*. *convoluta* demonstrates the effectiveness of hexane washing in reducing extraneous contamination by soil particles (Fig. 4). SEM–EDS point analysis of high As–Tl particles observed on the surface of the bryophytes indicated the abundance of As over Tl both before and after washing with hexane (Supplementary Files S1 and S2). The stoichiometric ratio between different elements (Si, S, Ca, Fe) indicates the mineral nature of these particles. The weight percentage detected by SEM–EDS in one of the samples analysed showed a reduction in As after washing from 18% to 3.25%, whereas Tl, although considerably lower, exhibited slight variations with both decreases and increases in local concentrations after washing (*e.g.* from 0.04 to 0.03% and from 0.22 to 0.34%).

## Discussion

### Elemental concentrations in bryophytes

Considerable variation was found in the elementome of bryophytes from Allchar, largely determined by site characteristics and anomalous concentrations of As and Tl in the substrate (Đorđević et al. 2021). This was particularly evident for As, with more than 60,000 µg g⁻^1^ in soil samples from the Southern Deposit, far exceeding values observed in previous studies (24,000 µg g⁻^1^; Jakovljević et al. 2025).

The general lack of cuticle, high surface-to-volume ratio, and high cation exchange capacity make bryophytes prone to inadvertent elemental uptake, which, together with slow release, results in anomalous concentrations when inhabiting these extreme habitats (Koz and Cevik 2014; Vásquez et al. 2019), as observed in *B*. *convoluta* collected in this part of the Allchar site, with As concentrations exceeding 2% in the plant tissue. Morphological characteristics, such as the absence of roots, at the same time, make bryophytes unsuitable for the application of hyperaccumulation criteria developed for vascular plants. Pending the development of bryophyte-specific criteria, the thresholds proposed for vascular plants (Van der Ent et al. 2013, 2021) were applied to identify hyperaccumulators in this group (Boyd et al. 2009; Lu et al. 2022). Of the 11 taxa analysed, seven had As concentrations exceeding the threshold set at 1000 µg g⁻^1^, with *Barbula ungiculata* showing higher levels than those found in the soil. To date, As accumulation has mainly been studied in aquatic species, able to accumulate much higher elemental concentrations than those present in the water, thus enabling their detection (Díaz et al. 2013; Suzuki et al. 2016; Sandhi et al. 2018). However, with the exception of *Scopelophila cataractae*, a Cu-accumulator species, with 1300 µg g⁻^1^ DW when cultivated in water containing only 0.005 mg/L As (Suzuki et al. 2016), the highest concentrations in most samples analysed were considerably below the threshold for accumulation proposed for vascular plants. Thus, in *W. fluitans*, a species known for its high As-tolerance, exposure to water treated for 24 h with 10 μM arsenate resulted in plant concentrations below 850 µg g⁻^1^ (Sandhi et al. 2018).

As with arsenic, high Tl concentrations in most bryophyte samples were associated with high Ti and Cr, indicating contamination with soil particles. In samples of *R*. *megapolitanum*, however, Tl concentrations exceeding the proposed threshold (100 µg g⁻^1^; Van der Ent et al. 2021) were found at relatively low Ti and Cr (47 and 3 µg g⁻^1^, respectively) and were higher than those found in the soil, suggesting potentially genuine hyperaccumulation. However, not much is known about its strategy for tolerating metal stress. Whereas bryophytes such as *Pohilia drummondii* and *Scorpiurum circinatum* (Brid.) M. Fleisch. & Loeske immobilise the metal(loid)s in the cell walls, preventing their entry into to the protoplast (Lang and Wernitznig 2011; Basile et al. 2012), in others, differences in cation exchange capacities and activities of plasma membrane transporters determine tolerance of these elements (Shakya et al. 2008). In *R*. *megapolitanum*, a pleurocarpous bryophyte, high accumulation was found despite lacking a large contact area with the substrate. The enrichment of As and Tl, being internal, with elemental accumulation in the protoplast, or through their immobilisation on the cell wall, was also found in *Dicranella howei*, a newly found species for North Macedonia (Sabovljević et al. 2026), where washing the samples with hexane removed considerable concentrations of Ti and Cr, efficiently eliminating contamination, however, the reduction in As and Tl remained quite limited.

The washing efficiency varied greatly among the samples, but appeared to be largely species-specific and could be attributed to several factors, primarily differences in ion exchange processes and elemental complexation involving organic functional groups in the bryophyte cell wall (Fernández-Martínez et al. 2021; Printarakul and Meeinkuirt 2025). Plant morphology is another key factor determining the level of contamination. The lowest concentrations of Ti and Cr, indicators of contamination, were found in *H. cupressiforme*, probably due to the position of the leaves, which are less movable when hydrated and thus less receptive to particles in the space between the ventral leaf parts and the stem surface in both wet and dry states. This makes cleaning the outer surface easier and access to the inner parts more difficult. This is rather significant since this species is widely used to determine atmospheric deposit pollution. In some species, growth form has a less pronounced effect on washing efficiency, as a considerable level was found for both pleurocarpous *R*. *megapolitanum* and acrocarpous *P*. *capillare*. The potential simultaneous hyperaccumulation of As and Tl observed in these two species may reflect differences in uptake pathways driven by their different growth types. In addition to variation in washing efficiency, in some species, elemental concentrations in washed samples exceeded those in unwashed samples. This is unexpected and could be due to rapid uptake and facilitated attachment of dissolved contaminants during the washing process, or the finer size fraction of the bryophyte material after washing, which increases the bulk density and thus the apparent concentrations when measured by MXRF. Additionally, mechanical disturbance or solvent interaction during washing can dislodge fine soil particles which may become re-trapped within the lower parts of bryophytes or bind to exposed cell wall functional groups.

### Distribution of ‘accumulated’ elements in bryophytes

Synchrotron μXRF analysis of selected bryophyte species from the Allchar site revealed the highest enrichment of As and Tl, with particularly anomalous concentrations in the lower parts of *B. convoluta*, attributed to the soil particulate contamination and difficulty in removing this with rinsing with demineralized water before the synchrotron analysis. Contamination was also indicated by the synchrotron µXRF map of titanium (Ti), an element with limited uptake and translocation in plants and commonly used as a soil contamination indicator (Cook et al. 2009; Van der Ent et al. 2018), which was present at more than 200 µg g⁻^1^ in the bryophyte samples before washing with hexane. Moreover, the co-localisation of As distribution hotspots with interspaces in the lower parts of* B*. *convoluta*, the co-localisation of particles rich in As and Tl across the entire sample of the same species, and the extreme abundance of both elements in Allchar soil, reaching up to 18 and 142 g kg⁻^1^ for Tl and As, respectively (Đorđević et al. 2021) further suggest contamination. The deposition of As and Tl on the bryophyte surface was also shown by SEM–EDS, with As greatly reduced after washing with hexane. In the same samples more subtle variation in Tl was observed, with a general increase in its local concentrations, suggesting internal distribution. Morphological specificities of the below-ground parts and dense habitus led to increased accumulation of other elements analysed, all likely related to soil contamination, to which field-collected plant material is often prone.

For Cu synchrotron μXRF analysis in *P. capillare* from Allchar, enrichment was found in both the lower parts and leaves, with depletion in the apical parts. With concentrations exceeding 100 µg g⁻^1^ in the leaves, Cu levels were much higher than in other species, but still below the threshold for hyperaccumulation suggested for vascular plants (300 µg g⁻^1^; Van der Ent et al. 2013, 2021). Although the concentrations were considerably higher compared to other species and accumulation was observed only for Cu, suggesting the potential of this species to remediate Cu-rich soil, the notably low concentrations of Cu in the samples determined by elemental analysis clearly indicate contamination of the synchrotron-analysed sample. An enrichment pattern similar to that of *P*. *capillare* from Allchar, with Cu accumulated mainly in the basal part of the shoot and concentrations decreasing towards the apical parts, was found in *P. capillare* from a copper mine site in Spain, under excessive Cu in the soil. Washing this sample with chloroform revealed a lipophilic (wax-like) barrier as the main exclusion mechanism, preventing metal uptake into the cells (Elvira et al. 2020), which could also underlie the negligible concentrations in the tissues of *P. capillare* from Allchar. No unique pattern was observed in Cu uptake in bryophytes (Shaw et al. 1987) or its toxicity (Nomura and Hasezawa 2011). In *S. cataractae*, a widely-known Cu-hyperaccumulating species, high concentrations of Cu affect plant development differently. Whereas lower concentrations of Cu in the substrate promote protonemal growth, high concentrations suppress gemmae development. This may represent a strategy to maintain expansion capacity when establishment occurs on substrates with high Cu loads that are nevertheless inhabited by this species. Such a pattern was not observed for other toxic elements (Nomura and Hasezawa 2011). The localisation of Zn varied between the samples, with species-specific accumulation already demonstrated by previous studies (Sabovljević et al. 2018). In taxa such as *H. cupressiforme* var. *lacunosum*, Zn accumulated mainly in the stem and basal part of the leaves, following the same principles observed in previous studies (Sabovljević et al. 2018), however, in this case, it was present in negligible concentrations further affected by contamination.

The apparent differences between synchrotron μXRF and SEM–EDS results can largely be attributed to the combined effects of analytical scale, sample preparation, and bryophyte morphology. SEM–EDS provides highly localised, surface-sensitive information and is particularly effective at detecting particulate material adhering to the plant surface, especially in morphologically complex structures such as dense rhizoids and compact basal tissues. In contrast, synchrotron μXRF integrates elemental signals over a larger interaction volume and is more sensitive to elements distributed within internal tissues, allowing partial discrimination between surface contamination and internal accumulation. In bryophytes with a dense habitus and extensive below-ground structures, soil particles may become tightly entrapped within rhizoid networks and intercellular spaces, making their complete removal impossible even with hexane washing. This leads to enhanced detection of lithogenic elements and co-associated metals by SEM–EDS, while μXRF may reveal internally localised elemental distributions. Consequently, the observed discrepancies between the two techniques reflect methodological complementarity rather than inconsistency, highlighting the strong influence of morphology-driven soil contamination in field-collected bryophyte material.

## Conclusions

This study conclusively demonstrates the accumulation of trace metals in selected bryophyte species from the Allchar site in North Macedonia, highlighting their potential as indicators of anomalous elemental concentrations in the environment. However, no clear pattern was observed, as expected, since many parameters can affect the presence of species, uptake, immobilisation, or accumulation of trace metals from the surface of highly contaminated substrates. The measured concentrations are significantly affected by the hexane washing procedure, underscoring the importance of standardising these during sample preparation. Despite the strong influence of extraneous contamination, the results revealed potential Tl hyperaccumulation in *R*. *megapolitanum* and *P*. *capillare*, and As hyperaccumulation in seven taxa, which have not been reported in previous studies. To establish thresholds for elemental accumulation specific to this group of plants, elementome analyses of a large number of bryophytes and analyses of elemental localisation within bryophyte tissues are needed. Finally, determining the chemical speciation of As and Tl inside bryophyte cells, using synchrotron X-ray Absorption Spectroscopy (XAS), could help to assess whether these elements are genuinely accumulated or are mineral deposits.

## Supplementary Information

Below is the link to the electronic supplementary material.

Supplementary file1 (DOCX 123 KB)

Supplementary file2 (DOCX 19 KB)

Supplementary file3 (DOCX 17 KB)

Supplementary file4 (DOCX 17 KB)

Supplementary file5 (DOCX 376 KB)

Supplementary file6 (DOCX 325 KB)

## Data Availability

The data that support this study will be shared upon reasonable request to the corresponding author.

## Abbreviations

EDS: Energy dispersive X-ray spectroscopy
SEM: Scanning electron microscopy
