# Supplementary material for: Trace element accumulation in bryophytes from Allchar (North Macedonia): contamination or genuine hyperaccumulation?
Source: Planta. 2026 Aug 5;264(3):84. doi: 10.1007/s00425-026-05092-x (PMC13437567; doi:10.1007/s00425-026-05092-x)
Supplement: Supplementary file 2 [file 425_2026_5092_MOESM2_ESM.docx]

**Trace element accumulation in bryophytes from Allchar (North Macedonia): contamination or genuine hyperaccumulation?**

Ksenija Jakovljević^1^, Marko S Sabovljević^2,3,4^, Katerina Bačeva Andonovska^5^,

Lucas Henquet^6^, Antony van der Ent^6*^

^1^Department of Ecology, Institute for Biological Research Siniša Stanković – National Institute of the Republic of Serbia, University of Belgrade, Serbia. [ksenija.jakovljevic@ibiss.bg.ac.rs](mailto:ksenija.jakovljevic@ibiss.bg.ac.rs)

^2^Institute of Botany and Botanical Garden, Faculty of Biology, University of Belgrade, Serbia. [marko@bio.bg.ac.rs](mailto:marko@bio.bg.ac.rs)

^3^Department of Plant Biology, Institute of Biology and Ecology, Faculty of Science, Pavol Jozef Šafárik University in Košice, Slovakia.

^4^Center of Plant Biotechnology and Conservation (CPBC), Belgrade, Serbia.

^5^Research Center for Environment and Materials, Macedonian Academy of Sciences and Arts, Skopje, North Macedonia. [kbaceva@manu.edu.mk](mailto:kbaceva@manu.edu.mk)

^6^Laboratory of Genetics, Wageningen University and Research, The Netherlands. [lucas.henquet@wur.nl](mailto:lucas.henquet@wur.nl); [antony.vanderent@wur.nl](mailto:antony.vanderent@wur.nl)

*Corresponding author: [antony.vanderent@wur.nl](mailto:antony.vanderent@wur.nl)

**Table S1**. Bryophytes collected at Allchar, with voucher numbers indicated (BEOU Bryo)*, soil samples collected, and samples used for synchrotron analysis.

| Voucher No. | Family | Taxon | Locality | Soil sample | Synchrotron analysis |
| --- | --- | --- | --- | --- | --- |
| 11114 | Pottiaceae | *Barbula convoluta* Hedw | Allchar, Central Deposit |  |  |
| 11127 | Pottiaceae | *Barbula convoluta* Hedw | Allchar, Central Deposit | + | + |
| 11128 | Pottiaceae | *Barbula convoluta* Hedw | Allchar, Central Deposit | + | + |
| 11131 | Pottiaceae | *Barbula convoluta* Hedw | Allchar, Central Deposit |  |  |
| 11140 | Pottiaceae | *Barbula convoluta* Hedw | Allchar, Southern Deposit | + |  |
| 11123 | Pottiaceae | *Barbula unguiculata* Hedw | Allchar, Crven Dol |  | + |
| 11129 | Pottiaceae | *Barbula unguiculata* Hedw | Allchar, Central Deposit | + |  |
| 11136 | Dicranellaceae | *Dicranella howei* Renauld & Cardot | Allchar, Southern Deposit |  |  |
| 11110 | Brachytheciaceae | *Homalothecium lutescens* (Hedw.) H.Rob. | Allchar, Central Deposit |  |  |
| 11111 | Hypnaceae | *Hypnum cupressiforme* Hedw. | Allchar, Central Deposit |  |  |
| 11115 | Hypnaceae | *Hypnum cupressiforme* Hedw. | Allchar, Central Deposit |  |  |
| 11122 | Hypnaceae | *Hypnum cupressiforme* Hedw. | Allchar, Crven Dol |  |  |
| 11124 | Hypnaceae | *Hypnum cupressiforme* Hedw. | Allchar, Crven Dol | + |  |
| 11126 | Hypnaceae | *Hypnum cupressiforme* Hedw. | Allchar, Central Deposit | + |  |
| 11130 | Hypnaceae | *Hypnum cupressiforme* Hedw. | Allchar, Central Deposit |  |  |
| 11133 | Hypnaceae | *Hypnum cupressiforme* Hedw. | Allchar, Central Deposit |  |  |
| 11134 | Hypnaceae | *Hypnum cupressiforme* Hedw. | Allchar, Central Deposit |  |  |
| 11141 | Hypnaceae | *Hypnum cupressiforme* Hedw. | Allchar, Southern Deposit | + |  |
| 11142 | Hypnaceae | *Hypnum cupressiforme* Hedw. | Allchar, Southern Deposit |  |  |
| 11146 | Hypnaceae | *Hypnum cupressiforme* Hedw. | Allchar, Southern Deposit |  |  |
| 11147 | Hypnaceae | *Hypnum cupressiforme* Hedw. | Allchar, Southern Deposit |  |  |
| 11149 | Hypnaceae | *Hypnum cupressiforme* Hedw. | Allchar, Southern Deposit |  |  |
| 11120 | Hypnaceae | *Hypnum cupressiforme* Hedw.var. *lacunosum* Brid. | Allchar, Crven Dol | + | + |
| 11135 | Bryaceae | *Ptychostomum capillare* Hedw. | Allchar, Central Deposit |  |  |
| 11138 | Bryaceae | *Ptychostomum capillare* Hedw. | Allchar, Southern Deposit | + | + |
| 11139 | Bryaceae | *Ptychostomum capillare* Hedw. | Allchar, Southern Deposit |  |  |
| 11144 | Bryaceae | *Ptychostomum capillare* Hedw. | Allchar, Southern Deposit |  |  |
| 11145 | Bryaceae | *Ptychostomum capillare* Hedw. | Allchar, Southern Deposit |  |  |
| 11148 | Bryaceae | *Ptychostomum capillare* Hedw. | Allchar, Southern Deposit |  | + |
| 11117 | Brachytheciaceae | *Rhynchostegium megapolitanum* (Web. et Mohr) B.S.G. | Allchar, Central Deposit | + |  |
| 11118 | Brachytheciaceae | *Rhynchostegium megapolitanum* (Web. et Mohr) B.S.G. | Allchar, Central Deposit |  |  |
| 11119 | Brachytheciaceae | *Rhynchostegium megapolitanum* (Web. et Mohr) B.S.G. | Allchar, Central Deposit – Crven Dol | + |  |
| 11121 | Brachytheciaceae | *Rhynchostegium megapolitanum* (Web. et Mohr) B.S.G. | Allchar, Crven Dol | + | + |
| 11125 | Brachytheciaceae | *Rhynchostegium megapolitanum* (Web. et Mohr) B.S.G. | Allchar, Crven Dol | + |  |
| 11132 | Brachytheciaceae | *Rhynchostegium megapolitanum* (Web. et Mohr) B.S.G. | Allchar, Central Deposit |  |  |
| 11137 | Brachytheciaceae | *Rhynchostegium megapolitanum* (Web. et Mohr) B.S.G. | Allchar, Southern Deposit |  |  |
| 11143 | Brachytheciaceae | *Rhynchostegium megapolitanum* (Web. et Mohr) B.S.G. | Allchar, Southern Deposit | + |  |
| 11112 | Pottiaceae | *Syntrichia ruraliformis* (Besch.) Cardot | Allchar, Central Deposit |  |  |
| 11113 | Pottiaceae | *Syntrichia ruralis* (Hedw.) F.Weber & D.Mohr | Allchar, Central Deposit |  |  |
| 11116 | Pottiaceae | *Syntrichia subpapilosissima* (W.A.Kramer) M.T.Gallego & J. Guerra | Allchar, Central Deposit |  |  |

* BEOU- acronym according to Index Herbariorum (2026)

Index Herbariorum (2026) Index Herbariorum: A Global Directory of Public Herbaria and Associated Staff. New York Botanical Garden. Published online: https://sweetgum.nybg.org/science/ih/ (05 May 2026).
