# Supplementary material for: Trace element accumulation in bryophytes from Allchar (North Macedonia): contamination or genuine hyperaccumulation?
Source: Planta. 2026 Aug 5;264(3):84. doi: 10.1007/s00425-026-05092-x (PMC13437567; doi:10.1007/s00425-026-05092-x)
Supplement: Supplementary file 3 [file 425_2026_5092_MOESM3_ESM.docx]

**Trace element accumulation in bryophytes from Allchar (North Macedonia): contamination or genuine hyperaccumulation?**

Ksenija Jakovljević^1^, Marko S Sabovljević^2,3,4^, Katerina Bačeva Andonovska^5^,

Lucas Henquet^6^, Antony van der Ent^6*^

^1^Department of Ecology, Institute for Biological Research Siniša Stanković – National Institute of the Republic of Serbia, University of Belgrade, Serbia. [ksenija.jakovljevic@ibiss.bg.ac.rs](mailto:ksenija.jakovljevic@ibiss.bg.ac.rs)

^2^Institute of Botany and Botanical Garden, Faculty of Biology, University of Belgrade, Serbia. [marko@bio.bg.ac.rs](mailto:marko@bio.bg.ac.rs)

^3^Department of Plant Biology, Institute of Biology and Ecology, Faculty of Science, Pavol Jozef Šafárik University in Košice, Slovakia.

^4^Center of Plant Biotechnology and Conservation (CPBC), Belgrade, Serbia.

^5^Research Center for Environment and Materials, Macedonian Academy of Sciences and Arts, Skopje, North Macedonia. [kbaceva@manu.edu.mk](mailto:kbaceva@manu.edu.mk)

^6^Laboratory of Genetics, Wageningen University and Research, The Netherlands. [lucas.henquet@wur.nl](mailto:lucas.henquet@wur.nl); [antony.vanderent@wur.nl](mailto:antony.vanderent@wur.nl)

*Corresponding author: [antony.vanderent@wur.nl](mailto:antony.vanderent@wur.nl)

**Table S2**. The concentrations of arsenic and thallium (in µg g⁻^1^) in single-sampled (*n* = 1) bryophyte taxa from the Allchar area under different treatments.

| **Taxon** | **Treatment** | ***n*** | **As** | **Tl** |
| --- | --- | --- | --- | --- |
| *Dicranella howei* | unwashed | 1 | 4890 | 0 |
|  | washed | 1 | 3050 | 33.8 |
| *Homalothecium lutescens* | unwashed | 1 | 15.7 | 1.02 |
|  | washed | 1 | 30.9 | 0 |
| *Hypnum cupressiforme* var. *lacunosum* | unwashed | 1 | 378 | 51.7 |
|  | washed | 1 | 2280 | 0 |
| *Syntrichia ruraliformis* | unwashed | 1 | 34 | 12.7 |
|  | washed | 1 | 32.2 | 15.9 |
| *Syntrichia ruralis* | unwashed | 1 | 36.6 | 10.5 |
|  | washed | 1 | 42.9 | 16.3 |
| *Syntrichia subpapilosissima* | unwashed | 1 | 68.8 | 19.6 |
|  | washed | 1 | 51.1 | 15 |
