# Supplementary material for: Trace element accumulation in bryophytes from Allchar (North Macedonia): contamination or genuine hyperaccumulation?
Source: Planta. 2026 Aug 5;264(3):84. doi: 10.1007/s00425-026-05092-x (PMC13437567; doi:10.1007/s00425-026-05092-x)
Supplement: Supplementary file 4 [file 425_2026_5092_MOESM4_ESM.docx]

**Trace element accumulation in bryophytes from Allchar (North Macedonia): contamination or genuine hyperaccumulation?**

Ksenija Jakovljević^1^, Marko S Sabovljević^2,3,4^, Katerina Bačeva Andonovska^5^,

Lucas Henquet^6^, Antony van der Ent^6*^

^1^Department of Ecology, Institute for Biological Research Siniša Stanković – National Institute of the Republic of Serbia, University of Belgrade, Serbia. [ksenija.jakovljevic@ibiss.bg.ac.rs](mailto:ksenija.jakovljevic@ibiss.bg.ac.rs)

^2^Institute of Botany and Botanical Garden, Faculty of Biology, University of Belgrade, Serbia. [marko@bio.bg.ac.rs](mailto:marko@bio.bg.ac.rs)

^3^Department of Plant Biology, Institute of Biology and Ecology, Faculty of Science, Pavol Jozef Šafárik University in Košice, Slovakia.

^4^Center of Plant Biotechnology and Conservation (CPBC), Belgrade, Serbia.

^5^Research Center for Environment and Materials, Macedonian Academy of Sciences and Arts, Skopje, North Macedonia. [kbaceva@manu.edu.mk](mailto:kbaceva@manu.edu.mk)

^6^Laboratory of Genetics, Wageningen University and Research, The Netherlands. [lucas.henquet@wur.nl](mailto:lucas.henquet@wur.nl); [antony.vanderent@wur.nl](mailto:antony.vanderent@wur.nl)

*Corresponding author: [antony.vanderent@wur.nl](mailto:antony.vanderent@wur.nl)

**Table S3**. The mean ± standard deviation and range of concentrations of chromium and titanium (in µg g^⁻1^) in bryophyte taxa from the Allchar area under different treatments. *n* represents the number of samples analysed.

| **Taxon** | **Treatment** | ***n*** | **Cr** | | **Ti** | |
| --- | --- | --- | --- | --- | --- | --- |
|  |  |  | **mean ± SD** | **min–max** | **mean ± SD** | **min–max** |
| *Barbula convoluta* | unwashed | 5 | 11.6 ± 5.52 | 3.42–18.7 | 339 ± 261 | 143–774 |
|  | washed | 5 | 52.2 ± 88.7 | 2.25–210 | 412 ± 247 | 187–813 |
| *Barbula unguiculata* | unwashed | 2 | 22.6 ± 17.5 | 10.3–35 | 243 ± 118 | 160–327 |
|  | washed | 2 | 49.9 ± 42.7 | 19.7–80.0 | 528 ± 152 | 421–636 |
| *Dicranella howei* | unwashed | 1 | 206 | 206 | 1775 | 1775 |
|  | washed | 1 | 31 | 31 | 438 | 438 |
| *Homalothecium lutescens* | unwashed | 1 | 6.0 | 6.0 | 85.9 | 85.9 |
|  | washed | 1 | 5 | 5 | 50 | 50 |
| *Hypnum cupressiforme* | unwashed | 17 | 9.87 ± 11.4 | 2.03–49.2 | 119 ± 104 | 55.5–448 |
|  | washed | 16 | 6.44 ± 5.48 | 2.34–25.7 | 95.0 ± 66.6 | 37.5–316 |
| *Hypnum cupressiforme* var*. lacunosum* | unwashed | 1 | 22.4 | 22.4 | 272.2 | 272.2 |
|  | washed | 1 | 69.0 | 69.0 | 897.9 | 897.9 |
| *Ptychostomum capillare* | unwashed | 4 | 13.4 ± 4.71 | 9.57–20.2 | 203 ± 66.8 | 145–299 |
|  | washed | 8 | 13.2 ± 5.38 | 7.5–21.4 | 225 ± 99.9 | 120–442 |
| *Rhynchostegium megapolitanum* | unwashed | 9 | 16.6 ± 8.65 | 2.42–28.1 | 209 ± 123 | 43.4–366 |
|  | washed | 11 | 13.0 ± 18 | 2.48–64.7 | 212 ± 261 | 46.6–956 |
| *Syntrichia ruraliformis* | unwashed | 1 | 13.9 | 13.9 | 178.6 | 178.6 |
|  | washed | 1 | 12.0 | 12.0 | 230.8 | 230.8 |
| *Syntrichia ruralis* | unwashed | 1 | 13 | 13 | 211 | 211 |
|  | washed | 1 | 11 | 11 | 209 | 209 |
| *Syntrichia subpapilosissima* | unwashed | 1 | 10.8 | 10.8 | 128.4 | 128.4 |
|  | washed | 1 | 7.9 | 7.9 | 89.3 | 89.3 |
