# Supplementary material for: Trace element accumulation in bryophytes from Allchar (North Macedonia): contamination or genuine hyperaccumulation?
Source: Planta. 2026 Aug 5;264(3):84. doi: 10.1007/s00425-026-05092-x (PMC13437567; doi:10.1007/s00425-026-05092-x)
Supplement: Supplementary file 5 [file 425_2026_5092_MOESM5_ESM.docx]

**Trace element accumulation in bryophytes from Allchar (North Macedonia): contamination or genuine hyperaccumulation?**

Ksenija Jakovljević^1^, Marko S Sabovljević^2,3,4^, Katerina Bačeva Andonovska^5^,

Lucas Henquet^6^, Antony van der Ent^6*^

^1^Department of Ecology, Institute for Biological Research Siniša Stanković – National Institute of the Republic of Serbia, University of Belgrade, Serbia. [ksenija.jakovljevic@ibiss.bg.ac.rs](mailto:ksenija.jakovljevic@ibiss.bg.ac.rs)

^2^Institute of Botany and Botanical Garden, Faculty of Biology, University of Belgrade, Serbia. [marko@bio.bg.ac.rs](mailto:marko@bio.bg.ac.rs)

^3^Department of Plant Biology, Institute of Biology and Ecology, Faculty of Science, Pavol Jozef Šafárik University in Košice, Slovakia.

^4^Center of Plant Biotechnology and Conservation (CPBC), Belgrade, Serbia.

^5^Research Center for Environment and Materials, Macedonian Academy of Sciences and Arts, Skopje, North Macedonia. [kbaceva@manu.edu.mk](mailto:kbaceva@manu.edu.mk)

^6^Laboratory of Genetics, Wageningen University and Research, The Netherlands. [lucas.henquet@wur.nl](mailto:lucas.henquet@wur.nl); [antony.vanderent@wur.nl](mailto:antony.vanderent@wur.nl)

*Corresponding author: [antony.vanderent@wur.nl](mailto:antony.vanderent@wur.nl)

**Supplementary File S1** SEM-EDS point analysis of high As-Tl particles observed on the surface of unwashed bryophytes.

| **Spectrum 1** | | |
| --- | --- | --- |
| Element | Wt% | Wt% Sigma |
| C | 16.32 | 0.75 |
| O | 44.39 | 0.48 |
| Si | 7.06 | 0.12 |
| Cl | 0.01 | 0.04 |
| K | 0.57 | 0.04 |
| Ca | 1.55 | 0.06 |
| Ti | 0.23 | 0.05 |
| Fe | 10.87 | 0.18 |
| As | 18.98 | 0.26 |
| Total: | 100.00 |  |

| **Spectrum 2** | | |
| --- | --- | --- |
| Element | Wt% | Wt% Sigma |
| C | 9.88 | 0.80 |
| O | 42.61 | 0.45 |
| Si | 3.85 | 0.11 |
| S | 0.11 | 0.04 |
| Cl | 0.00 | 0.04 |
| K | 0.16 | 0.04 |
| Ca | 1.04 | 0.05 |
| Fe | 13.86 | 0.20 |
| As | 28.50 | 0.33 |
| Total: | 100.00 |  |
