# Supplementary material for: Trace element accumulation in bryophytes from Allchar (North Macedonia): contamination or genuine hyperaccumulation?
Source: Planta. 2026 Aug 5;264(3):84. doi: 10.1007/s00425-026-05092-x (PMC13437567; doi:10.1007/s00425-026-05092-x)
Supplement: Supplementary file 6 [file 425_2026_5092_MOESM6_ESM.docx]

**Trace element accumulation in bryophytes from Allchar (North Macedonia): contamination or genuine hyperaccumulation?**

Ksenija Jakovljević^1^, Marko S Sabovljević^2,3,4^, Katerina Bačeva Andonovska^5^,

Lucas Henquet^6^, Antony van der Ent^6*^

^1^Department of Ecology, Institute for Biological Research Siniša Stanković – National Institute of the Republic of Serbia, University of Belgrade, Serbia. [ksenija.jakovljevic@ibiss.bg.ac.rs](mailto:ksenija.jakovljevic@ibiss.bg.ac.rs)

^2^Institute of Botany and Botanical Garden, Faculty of Biology, University of Belgrade, Serbia. [marko@bio.bg.ac.rs](mailto:marko@bio.bg.ac.rs)

^3^Department of Plant Biology, Institute of Biology and Ecology, Faculty of Science, Pavol Jozef Šafárik University in Košice, Slovakia.

^4^Center of Plant Biotechnology and Conservation (CPBC), Belgrade, Serbia.

^5^Research Center for Environment and Materials, Macedonian Academy of Sciences and Arts, Skopje, North Macedonia. [kbaceva@manu.edu.mk](mailto:kbaceva@manu.edu.mk)

^6^Laboratory of Genetics, Wageningen University and Research, The Netherlands. [lucas.henquet@wur.nl](mailto:lucas.henquet@wur.nl); [antony.vanderent@wur.nl](mailto:antony.vanderent@wur.nl)

*Corresponding author: [antony.vanderent@wur.nl](mailto:antony.vanderent@wur.nl)

**Supplementary File S2** SEM-EDS point analysis of high As-Tl particles observed on the surface of the bryophytes after washing with hexane.

| **Spectrum 5** | | |
| --- | --- | --- |
| Element | Wt% | Wt% Sigma |
| C | 35.68 | 0.47 |
| O | 45.85 | 0.41 |
| Si | 2.41 | 0.06 |
| S | 4.65 | 0.07 |
| Cl | 0.02 | 0.02 |
| K | 0.13 | 0.03 |
| Ca | 7.51 | 0.09 |
| Ti | 0.16 | 0.03 |
| As | 3.25 | 0.09 |
| Tl | 0.34 | 0.19 |
| Total: | 100.00 |  |

| **Spectrum 6** | | |
| --- | --- | --- |
| Element | Wt% | Wt% Sigma |
| C | 26.59 | 0.58 |
| O | 41.71 | 0.41 |
| Si | 5.03 | 0.10 |
| S | 0.38 | 0.04 |
| Cl | 0.00 | 0.03 |
| K | 0.43 | 0.04 |
| Ca | 3.79 | 0.07 |
| Fe | 6.44 | 0.13 |
| As | 15.61 | 0.21 |
| Total: | 100.00 |  |
